# Large-scale analysis of the evolutionary histories of phosphorylation motifs in the human genome

**DOI:** 10.1186/s13742-015-0057-6

**Published:** 2015-05-06

**Authors:** Hisayoshi Yoshizaki, Shujiro Okuda

**Affiliations:** 1 Department of Pathology I, Kanazawa Medical University, 1-1 Daigaku, Uchinada, Ishikawa, 920-0293 Japan; 2 Department of Biomedical Sciences, College of Life Sciences, Ritsumeikan University, 1-1-1 Noji-higashi, Kusatsu, Shiga 525-0058 Japan; 3 Graduate School of Medical and Dental Sciences, Niigata University, 1-757 Asahimachi-dori, Chuo-ku, Niigata 951-8510 Japan

**Keywords:** Phosphorylation motif, Comparative evolutionary analysis, Kinase

## Abstract

**Background:**

Protein phosphorylation is a post-translational modification that is essential for a wide range of eukaryotic physiological processes, such as transcription, cytoskeletal regulation, cell metabolism, and signal transduction. Although more than 200,000 phosphorylation sites have been reported in the human genome, the physiological roles of most remain unknown. In this study, we provide some useful datasets for the assessment of functional phosphorylation signaling using a comparative genome analysis of phosphorylation motifs.

**Findings:**

We described the evolutionary patterns of conservation of these and comparative genomic data for 93,101 phosphosites and 1,003,756 potential phosphosites in human phosphomotifs, using 178 phosphomotifs identified in a previous study that occupied 69% of known phosphosites in public databases. Comparative genomic analyses were performed using genomes from nine species from yeast to humans. Here we provide an overview of the evolutionary patterns of phosphomotif acquisition and indicate the dependence on motif structures. Using the data from our previous study, we describe the interaction networks of phosphoproteins, identify the kinase substrates associated with phosphoproteins, and perform gene ontology enrichment analyses. In addition, we show how this dataset can help to elucidate the function of phosphomotifs.

**Conclusions:**

Our characterizations of motif structures and assessments of evolutionary conservation of phosphosites reveal physiological roles of unreported phosphosites. Thus, interactions between protein groups that share motifs are likely to be helpful for inferring kinase-substrate interaction networks. Our computational methods can be used to elucidate the relationships between phosphorylation signaling and cellular functions.

**Electronic supplementary material:**

The online version of this article (doi:10.1186/s13742-015-0057-6) contains supplementary material, which is available to authorized users.

## Data description

### Utility of the dataset

Protein phosphorylation has an important role in a wide variety of cellular functions [1], and previous large-scale mass spectrometry studies have identified >100,000 phosphosites [2,3]. These phosphosites mostly represent modifications with unknown physiological functions, precluding identification of which ones are physiologically important. Nonetheless, 518 protein kinases have been reported in the human genome and, because various kinases are targeted to specific sequence motifs in the surrounding regions of phosphosites, such phosphorylation motifs have been extensively characterized [4]. Here, we have determined the functions of phosphorylation signaling pathways in cellular processes. We have also investigated the relationships between 178 phosphomotifs and cellular functions, and evolutionary conservation [5]. Our analyses indicate that highly conserved phosphomotifs are likely to be involved in similar signaling networks with functionally important roles. We describe the sequences and evolutionary conservation of 93,101 known phosphosites and 1,003,756 potential phosphosites from the human genome (Additional file 1 and Raw_Data_All_Motif_Seq.txt in GigaDB [6]). This information is expected to be helpful for linking phosphorylation signaling networks to physiological functions and for assessing functional importance. Therefore, we provide information about the kinases that phosphorylate them, the interaction networks of proteins with the same motif, and we find the associations between the motifs and the biological functions. We show that this dataset can help to elucidate the function of phosphomotifs and their role in cellular signaling by showing how they evolved. Furthermore, we show information about the evolutionary conservation of phosphosites with known kinase-substrate relationships, and the ortholog conservation of each kinase. Finally, we show that the evolutionary conservation of phosphomotifs is not likely to be correlated with the ortholog conservation of the kinases.

### Definition of the phosphomotif conservation index

In a previous study, we identified 178 phosphomotifs [5] and investigated the evolutionary conservation of the phosphosites in each motif to elucidate the physiological roles of the identified motifs. To evaluate evolutionary conservation, we selected model organisms with rich genome information, as follows: *Saccharomyces cerevisiae*, *Schizosaccharomyces pombe*, *Caenorhabditis elegans*, *Drosophila melanogaster*, *Danio rerio*, *Canis familiaris*, *Mus musculus*, *Pan troglodytes*, and *Homo sapiens*. Subsequently, the phosphorylation sites defined by Beltrao *et al*. [7] and Minguez *et al*. [8] were extracted, along with those from the PHOSHIDA database [9] and the dbPTM 3.0 database [10], including databases such as Phospho.ELM [11], HPRD [12], and the PhosphoSitePlus database [13,14], which were downloaded on 12 November 2014 (Additional file 1). Orthologous gene sets were generated from the KEGG ortholog clusters (OCs) for the nine species [15]. For each OC, multiple sequence alignments were constructed using the freely available, rapid, and reliable multiple alignment tool MAFFT [16]. MAFFT was run with the “-auto” option for the automatic selection of optimal parameters. Sequence regions with precisely matched known phosphomotifs were then identified from all of the species, and species conservation was evaluated with respect to known and potential phosphorylation sites. Potential phosphosites were defined as all serine, threonine, and tyrosine residues (STY residues) in human proteins harboring known phosphosites. The conservation rates were then calculated for all of the known and potential phosphosites in the nine species, and the conservation rates for each phosphomotif were defined as the number of phosphosites conserved in a species divided by the number of phosphosites observed in the human genome. To determine the differences between the known and potential phosphosites, conservation indices (CIs) were calculated as the sum of the difference between the conservation rate of a phosphosite in a motif (C) and the reference conservation rate (R) of the corresponding amino acid residue obtained from all human proteins. CIs were calculated using the following equation:

$$ CI={\displaystyle \sum_{q\ \in G}\left({C}_q-{R}_q\right)} $$

where G denotes the set of genomes used in the study, q denotes the index of a genome selected from G, and C_q_ and R_q_ are the conservation and reference conservation rates in q, respectively.

**Figure 1:**
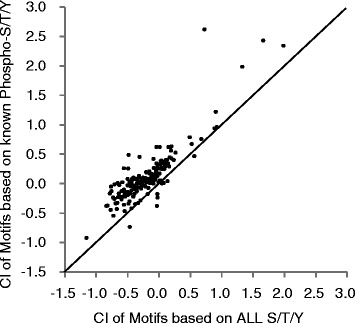
Scatterplot of the CIs of known phosphosites against those of all phosphorylation residues in the human genome. CIs of motifs with known phosphosites and all serine/threonine/tyrosine residues were plotted: the correlation coefficient is 0.865. The solid line indicates y = x. See also Data2-1, Data2-2, and Data2-3 in Additional file 2.

**Figure 2:**
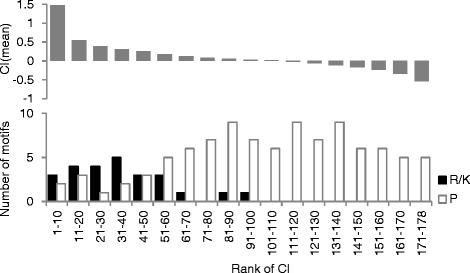
Histogram of phosphomotifs, including R-x-x-S/T and S/T-P patterns based on CIs. Frequency distribution of CIs for the phosphomotifs including S/T-P and basic amino acid residues at the N-terminals was calculated (see Additional file 3). Each class of the frequency distribution includes a successive group of ten phosphomotifs ordered by CI (rank of CI). Average CIs in a class are presented as plots in the upper panel. The lower panel shows the number of phosphomotifs including R/K-x-x-S/T, R/K-x-x-x-S/T, or R/K-x-S/T (black) and S/T-P (white) patterns for ranked CI. See also Additional file 3.

The CIs in each phosphomotif were compared between known phosphosites and all STY residues. Importantly, these comparisons support the recently reported tendencies of highly phosphorylated sites to be more conserved than less phosphorylated sites [17]. Moreover, known phosphosites showed higher conservation in most phosphomotifs than in all of the STY residues (Figure 1 and Additional file 2). However, some phosphosites showed similar or lower CIs than known phosphosites. Moreover, these poorly conserved phosphomotifs were likely to include S/T-P amino acid sequences. In contrast, arginine-rich sequences in highly conserved phosphomotifs, such as R-X-X-S/T, tended to reside in the anterior region of the motif (Figure 2 and Additional file 3). Our analyses of the motif structures surrounding phosphosites suggest highly variable evolutionary conservation of phosphomotifs and dependence on motif structures.

### Evolutionary conservation and expansion of kinases

**Figure 3:**
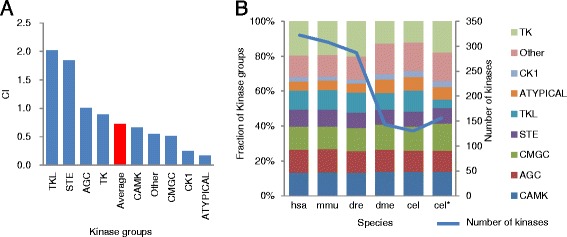
Evolutionary expansion and conservation of kinases and respective substrates. (**A**) Evolutionary conservation of substrates of kinase groups: CIs for each kinase group defined in PhosphoSitePlus [13] were calculated using the same method as the CIs of phosphomotifs (see the section of the definition of phosphomotif CIs). See also Data5-1 in Additional file 5. (**B**) Evolutionary expansions of kinase groups: 371 kinases were classified into nine kinase groups and were extracted from PhosphoSitePlus and KEGG BRITE databases. Orthologs of the kinases in the genomes hsa, mmu, dre, dme, and cel were assigned by the KEGG SSDB homology database. See also Additional file 6. Fractions of kinase groups for each genome obtained from KEGG SSDB were calculated. In addition, the fraction that was calculated for the kinases classified in WormBase used in the study by Lehmann et al. is indicated by an asterisk [28,29]. The blue line indicates the number of kinase genes. The assignment of kinase groups was performed using PhosphoSitePlus [13,14]. hsa, *Homo sapiens*; mmu, *Mus musculus*; dre, *Danio rerio*; dme, *Drosophila melanogaster*; cel, *Caenorhabditis elegans*.

Identification of the kinases involved in the phosphorylation of the motifs we identified allows inferences to kinases that bind protein substrates with these motif structures. To investigate the correlations between CIs and the kinase families of these motifs, data describing the relationships between kinases and substrates were extracted from PhosphoSitePlus [13,14]. Next, protein kinases that phosphorylate S/T-P, R-X-X-S/T, and R-X-S/T sequence patterns were identified. Most S/T-P sites (86%) were phosphorylated by kinases in the CMGC family, and more than 80% of the phosphomotifs harboring anterior arginine residues and lacking proline were phosphorylated by kinases of AGC and CAMK families (Table 1 and Additional file 4). These results suggest that ST-P is subject to phosphorylation by CMGC kinases, whereas R-X-X-S/T sites are more likely to be phosphorylated by AGC kinases. Thus, the phosphorylation substrates of AGC may be more highly conserved than those of the CMGC family. To confirm this hypothesis, we have calculated the evolutionary conservation of phosphosites with the reported kinase-substrate binding and CIs for each kinase family (Figure 3A and Additional file 5). In these analyses the substrate conservation of AGC kinases was greater than that of the CMGC kinases in all of the species.

**Table 1:** **Relationships between phosphorylation motifs and protein kinases**

Motif id	Motif sequence	Number of substrates	Kinase classification	1st		2nd		3rd	
12	S/T-P	3671	Group	CMGC	84%	AGC	6%	ATYPICAL	4%
			Family	MAPK	41%	CDK	35%	GSK	6%
166	R-X-S/T	1873	Group	AGC	65%	CAMK	10%	Other	10%
			Family	PKA	33%	PKC	19%	AUR	7%
173	R-X-X-S/T	2908	Group	AGC	61%	CAMK	21%	CMGC	8%
			Family	PKA	24%	PKC	13%	AKT	10%

The top three fractions of kinase groups and families are shown.

A previous report has shown that numbers of CMGC kinases have occurred during the early evolution of vertebrates [18]. Thus, to investigate the correlations of the CMGC kinase substrate conservation with the evolutionary expansion of kinases, changes in the numbers of kinases in the kinase groups were calculated using orthologs of kinases defined in KEGG. The proportion of AGC and CMGC kinases among all of the kinases did not differ between human and worm genomes (Figure 3B and Additional file 6), suggesting that numbers of AGC and CMGC kinases increased in vertebrates. Hence, conservation of phosphosites may reflect the types of kinases rather than the evolutionary changes in the numbers of expressed kinases. Thus, to facilitate the development of phosphomotif prediction tools, such as Scansite and Netphorest [19,20], we determined the evolutionary conservation of these phosphosites and defined the kinase-substrate relationships, CIs for each kinase family, and kinase orthologs.

### Interaction networks of proteins with assigned phosphorylation motifs

Interactions between proteins with the same motif were more likely than reconstructed interactions between randomly selected proteins, allowing enrichment of proteins with similar physiological functions [5]. Hence, identification of protein networks with the same motifs may facilitate characterizations of phosphorylation interaction networks based on kinase-substrate relationships and may be used to determine the ensuing physiological functions. To identify the associations between motif-associated proteins, data describing intermolecular interactions were downloaded from BioGRID (2.0.58) [21] and STRING (v8.2) [22], and the interactions of proteins with known motifs were extracted. The free open-source software application Cytoscape [23] was then used to visualize and analyze networks [24] and to construct network visualizations of our data (phospho-signal_network.cys in GigaDB [6]). Network visualization required the use of Cytoscape version 3.0 or above.

### Gene ontology enrichment analysis

We have previously identified [5] the likely functional correlations between a wide variety of phosphomotifs, warranting the characterization of phosphomotifs with functional categories, such as gene ontology (GO), to confirm the physiological functions of the ensuing phosphorylation signaling. Thus, correlations between extracted phosphorylation motifs and specific physiological protein functions were identified here using functional enrichment analysis based on GO (Additional file 7). In these analyses, GO annotations were extracted for human proteins with known phosphorylation sites. Subsequently, annotations at the known motif level were assigned on the basis of the GO biological processes for proteins with the motif, and motif functions were identified using enrichment analysis with GoMiner [25]. Significant GO annotations were extracted with cutoffs of FDR = 0.01 and p < 0.01.

## Availability of supporting data

Datasets supporting the results of this study are available in the *GigaScience* repository, GigaDB [6]. The data derived from PhosphoSitePlus is licensed under a Creative Commons Attribution-NonCommercial-ShareAlike 3.0 Unported License.

## Additional files

Additional file 1: **List of known phosphorylation sites.** Data 1: List of phosphorylation sites. Human phosphorylation sites for the 13,347 proteins used in this study. Information about the papers or databases for the phosphosites is indicated by the references: “B” [7], “DP” [10], “MG” [8], “PS” [9], and “PSP” [14]. Additional file 2: **Data used to generate Figure**1**.** Data 2–1: All of the defined phosphomotifs, including phosphomotif patterns and CIs of phosphomotifs possessing known phosphorylation residues and all STY residues. Data 2–2: Data used for calculation of CIs based on all STY residues. The conservation rate C is defined as the conservation fraction of each species relative to that for all human phosphosites that were grouped as a motif. Column C-R indicates the difference between C and the reference conservation rate R of the corresponding amino acid residue with the central residues of a motif (S/T/Y/ST) from all human proteins. CIs were calculated as the sum of values in the C-R column, and the CI in this table reflects CIs based on all STY residues in the human genome. Data 2–3: This table is the same as Data 2–2 except that the CI calculations are based on known phosphosites. Additional file 3: **Data used to generate Figure**2**.** Data 3: Defined phosphomotifs are presented in descending order of CIs and the successive sets of ten motifs from the top are grouped. Average CIs were then calculated for each group, and R/K and P types were counted. The R/K type is defined as motifs with R or K residues located at positions from −4 to −2 (blue), and the P type is defined as motifs with P residues located at position 1 (black). Additional file 4: **Data used to generate Table**1. Data 4–1: Relationships between phosphorylation motifs and respective protein kinases. Numbers of known phosphosites in each phosphomotif are shown. Among these phosphosites the numbers of phosphosites with defined kinase-substrate relationships in public databases are shown and the fractions of kinase families are calculated: the top five fractions are shown and phosphomotifs with fewer than ten kinase-substrate relationships are omitted. Data 4–2: Relationships between the phosphorylation motifs and their respective protein kinases. Annotations of kinase group names were determined on the basis of known kinase-substrate relationships from the phosphorylation database PhosphositePlus [13,14]. Data 4–3: List of kinase groups. Additional file 5: **Data used to generate Figure** 3**A.** Data 5–1: Data for calculation of CIs for kinase groups. CIs were calculated for each kinase group using the method that was used to calculate the CIs in phosphomotifs. Ref indicates a conservation rate of all serine and threonine residues of the human genome. Data 5–2: List of phosphorylation substrates and respective kinases. Uniprot identifiers for phosphorylation substrates, respective kinases, positions, residues, and sequences of phosphosites are listed. The conservation parameter indicates the most distant species (KEGG three letter organism code) from human with a conserved phosphosite. Kinase groups are defined according to PhosphoSitePlus [13,14]. Additional file 6: **Data used to generate Figure **3**B.** Data 6: Evolutionary expansions of kinase groups among 371 kinases, nine kinase groups were classified and extracted from PhosphoSitePlus [13,14] and KEGG BRITE [26]. Orthologs of the kinases in the genomes hsa, mmu, dre, dme, and cel were assigned according to the KEGG SSDB homology database [27] and the kinase groups were assigned according to PhosphoSitePlus. hsa, *Homo sapiens*; mmu, *Mus musculus*; dre, *Danio rerio*; dme, *Drosophila melanogaster*; cel, *Caenorhabditis elegans*. Additional file 7: **Clustering of enriched GO annotation profiles in phosphomotifs.** Biological processes in the GO annotations were used and profiles were clustered based on Euclidean distance using Ward’s method. Pink indicates that annotations were present; black indicates the absence of annotations. Functional categories listed on the left hand side of the table are based on functions that are associated with GO biological processes. See also the GoMiner directory in GigaDB [6].

## Abbreviations

GO: Gene Ontology
KEGG: Kyoto Encyclopedia of Genes and Genomes
OC: Ortholog Cluster
STRING: Search Tool for Recurring Instances of Neighboring Genes
